# Parotid Gland Solitary Fibrous Tumor Presenting as a Long Duration Mass: A Case Report

**DOI:** 10.1155/2022/2097634

**Published:** 2022-02-24

**Authors:** Khadijah Abdulhaleem, Mohammad Anas Dababo, Eyas Othman

**Affiliations:** ^1^Department of Central Military Laboratory and Blood Bank, Prince Sultan Military Medical City, Riyadh, Saudi Arabia; ^2^Department of Pathology and Laboratory Medicine, King Faisal Specialist Hospital and Research Centre, Riyadh, Saudi Arabia; ^3^Department of Otolaryngology, King Faisal Specialist Hospital and Research Centre, Riyadh, Saudi Arabia

## Abstract

The solitary fibrous tumor (SFT) is a tumor of uncertain histogenesis, affecting deep soft tissues, particularly the pleura (pulmonary) and extrapulmonary sites including thighs, retroperitoneum, other serosal surfaces, and cranial and spinal meninges. SFT and hemangiopericytoma are now considered the same entity, with general agreement on referring to this group of tumors as “SFT.” SFTs are generally benign tumors with small subsets of malignant ones. Moreover, they are well-circumscribed with a good prognosis after surgical resection. SFTs are uncommon in the head and neck and are quite rare in the parotid gland region. Here, we present a case of a 48-year-old female with SFT of the parotid gland region; the diagnosis was confirmed by positive immunohistochemical staining for Bcl-2, CD34, and STAT6. STAT6 immunohistochemistry is sensitive and specific for SFTs.

## 1. Introduction

A solitary fibrous tumor (SFT) is a rare soft tissue spindle cell neoplasm primarily affecting the pleura. In 1931, Klemperer and Rabin first described the tumor as a primary neoplasm involving the pleura and classified plural tumors into diffused and localized types [1]. Lietaud, in 1767, and Wagner, in 1870, were the first to report SFT in the pleura and noted the localized nature of the lesion [2]. In recent years, SFT has been reported in a variety of extrapleural sites. SFT in the head and neck region accounts for approximately 10-15% of all SFT cases [3]. The most common sites affected are the nasal, paranasal, nasopharynx, larynx, and oral cavities and the orbit [4]. Other head and neck sites include major salivary glands, especially the parotid gland, where 35 cases have been reported in the English literature. Parotid SFT was first described in1995 by Hanau and Miettinen [5]. Since SFTs of the parotid gland are unusual, these tumors may be misdiagnosed as other more common tumors in salivary glands. Herein, we report an additional case of SFT of the parotid gland without recurrence in a twelve-month follow-up.

## 2. Case Presentation

A 48-year-old female presented with a painless swelling in the right parotid gland region for 12 years. The mass was slowly increasing in size, especially over the past several months. Moreover, there was no history of constitutional symptoms. Her past medical/surgical history was unremarkable. Physical examination showed a right-sided parotid region, nontender, firm, immobile, smoothly countered mass without overlying erythema. There was no facial nerve paralysis or paresthesia. Cervical lymph nodes were not palpable.

**Figure 1:**
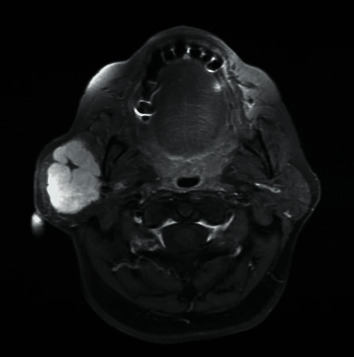
An axial MRI image demonstrating a lobulated lesion centered within the superficial lobe parotid gland (right side).

Magnetic resonance imaging (MRI) for the head and neck showed a large mildly lobulated lesion centered within the superficial lobe of the right parotid gland with extension anterior to the right mandibular ramus into the masticator space (Figure 1). The mass in the parotid gland measured approximately 4.3 × 3.2 × 5.2 cm in the anteroposterior, transverse, and craniocaudal dimensions, respectively. The tumor has a T2 hyperintense signal and a T1 intermediate signal with avid enhancement. The tumor is relatively well-marginated with preservation of the surrounding parotid parenchyma. There is no focal lesion in the left parotid gland or the submandibular glands.

Fine-needle aspiration (FNA) was performed, which yielded mainly blood aspirate and no diagnostic material. The patient underwent a right total parotidectomy without complications.

**Figure 2:**
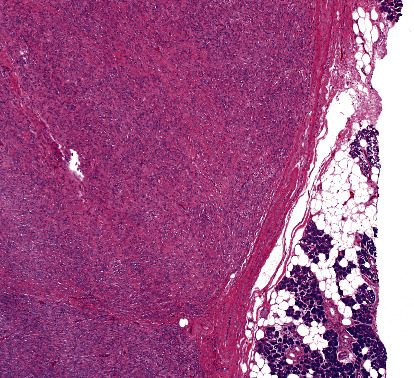
The lesion with well-defined border and showed a thin fibrous capsule. Unremarkable salivary gland tissue noted in the periphery (H&E, ×10).

**Figure 3:**
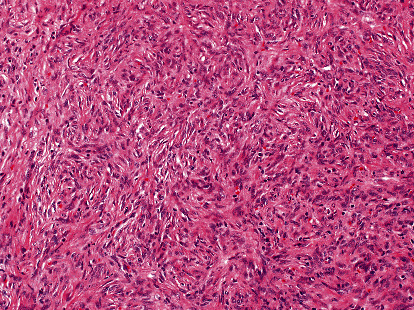
The tumor with spindle-shaped cells without a defined architectural pattern forming short fascicles with interspersed thin collagen bundles (H&E, ×20).

**Figure 4:**
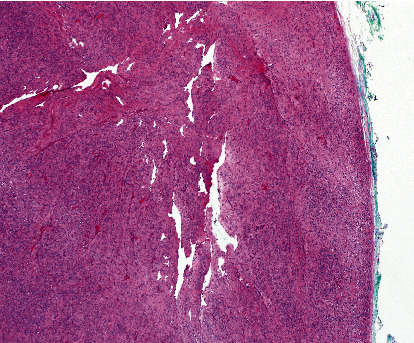
Delicate thin-walled vascular spaces, with hemangiopericytoma-like vasculature, were observed at the periphery (H&E, ×10).

Macroscopically, the parotid gland measures 7.0 × 5.0 × 3.0 cm. Serial sections revealed a multilobulated nodular, pale to tan, rubbery mass measuring 6.5 × 5.0 × 2.3 cm, identified on the inked margin. Microscopically, the tumor was relatively well-defined and showed a thin fibrous capsule (Figure 2). There was unremarkable salivary gland parenchyma in the background, with the tumor present at the inked margin of the sample. The tumor showed a normal salivary duct at the edge of the lesion. The neoplasm was composed of a lobulated proliferation of spindle-shaped cells without a defined architectural pattern forming short fascicles with interspersed thin collagen bundles (Figure 3). The spindle cells were monotonous, rounded to oval nuclei with coarse nuclear chromatin distribution and tapering eosinophilic cytoplasm. Delicate thin-walled vascular spaces, with hemangiopericytoma-like vasculature, were observed at the periphery (Figure 4). A few lymphocytes and scattered mast cells were present among the neoplastic cells. Mitosis, necrosis, cellular pleomorphism, or other signs of malignancy were absent.

**Figure 5:**
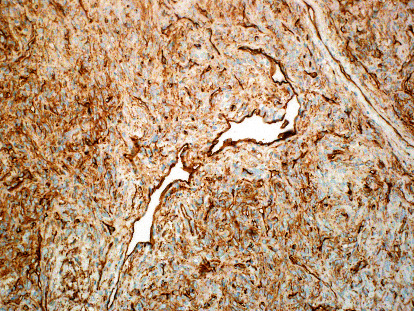
A diffuse positive immunoreactivity against CD34 (×20).

**Figure 6:**
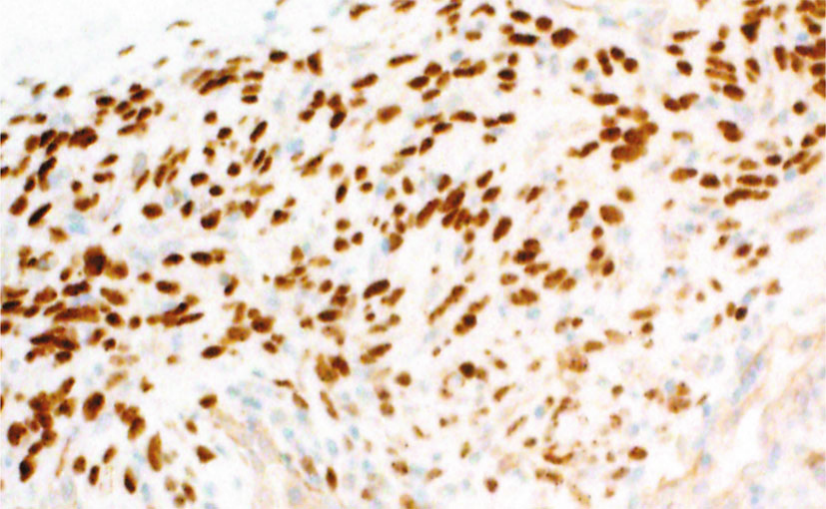
Positive immunoreactivity against STAT6, nuclear type positivity (×20).

Immunohistochemical studies were conducted using cytokeratin AE1/AE, EMA, S100, CD34, CD31, CD21, CD23, SMA, Bcl2, CD99, and STAT6. The tumor cells were strongly positive for Bcl2 and CD34 (Figure 5) and showed focal membrane positivity for CD99 and diffuse nuclear positivity for STAT6 (Figure 6), whereas they were negative for cytokeratin AE1/AE3, EMA, S-100 protein, CD21, CD23, SMA, and CD31. These results confirmed our diagnosis of SFT and ruled out other differential diagnoses such as spindle cell carcinoma, spindle cell myoepithelioma, schwannoma, and monophasic synovial sarcoma.

The patient has been followed up for twelve months, with no signs of recurrence or complications.

## 3. Discussion

SFT is a rare neoplasm that involves the intrathoracic and extrathoracic sites. SFTs are derived from pluripotent fibroblastic cells [6]. These tumors of uncertain behavior, locally aggressive, rarely metastasizing, can be malignant under the 2020 WHO classification. SFTs most commonly arise from the intrathorax site, especially affecting the pleura, which account for less than 5% of all neoplastic diseases [6]. Extrathorax SFTs involve different sites, such as soft tissue, peritoneum, pelvis, genitourinary, and head and neck regions. The salivary gland, especially the parotid gland, is one of the rarely involved regions. Only thirty-five cases of SFT in the parotid gland have been reported in the English literature [2, 4, 5, 7–34]. In 2012, Bauer et al. reviewed the literature for the first 22 cases of SFT in the parotid gland, three of which were malignant [2]. From 2012 to 2016, seven more additional cases were published [25–30]. In 2019, Lim et al. reviewed all reported cases of SFT in the major salivary glands, including the parotid area, from 2004 to 2018 [33]. Thus, including our case, only 36 cases have been reported to date. The pathologists must consider SFT in the differential diagnosis of spindle cell-rich lesions affecting the parotid gland such as spindle cell myoepithelioma, schwannoma, spindle cell carcinoma, low-grade myofibroblastic sarcoma, and monophasic synovial sarcoma. However, when suspected based on morphology, the diagnosis can be relatively straightforward, especially with using immunohistochemical stains. Parotid SFT is most often present as a well-defined, palpable, painless, and slow-growing mass, with a median size of 4.4 cm [26, 28]. In their review, Sousa et al. [26] have indicated a slight male predominance (1.2 : 1 M : F ratio) and a mean age of 49.8 years. SFT affects patients of all age groups ranging from 11 to 79 years and is more common in the left parotid gland [26]. Microscopically, the tumor is composed of spindle to ovoid cells arranged in a short fascicular pattern. The cells have a moderate amount of eosinophilic cytoplasm, centrally located nuclei with open, vesicular nuclear chromatin, and indistinct nucleoli. The stroma shows medium-sized hemangiopericytoma-like vessels, in addition to a collagen matrix, myxoid changes, and some inflammatory cells such as mast cells [26]. Immunohistochemical stains are important to confirm the diagnosis. SFT is positive for vimentin, CD34, Bcl2, and CD99 [26]. CD34 is important and a sensitive marker for SFT, although it may not be positive in all cases [2].

Furthermore, diffuse nuclear expression of STAT6 has been found to be a highly sensitive and specific immunohistochemical marker for SFT [31]. Recently, several studies have detected a recurrent intrachromosomal fusion between the NAB2 and STAT6 genes on chromosome 12 in SFT [31]. These gene fusions became pathognomonic for SFT. To our knowledge, this is the third case report that confirms the diagnosis of parotid SFT by diffuse nuclear positivity of STAT6 by immunohistochemical study. Complete local surgical excision with negative margins is the most important prognostic factor in SFT, and a long-term follow-up is recommended [27]. Similar to our case, Ganly et al. [32] have suggested that the patients with positive surgical margins or whose tumors have a malignant component benefit from postoperative radiotherapy. However, few reports have documented this issue and their follow-up periods were short. In our case, there was a positive microscopic surgical margin. We presented the case to the Head and Neck Oncology Committee and decided that no adjuvant therapy was necessary. Bauer et al. [2] have suggested that the cases of parotid gland SFT with positive margins showed no recurrence to date and follow-up was not required. In our case of parotid SFT, patient age, sex, duration of symptoms, size, and immunohistological findings were consistent with previously reported ranges.

In conclusion, SFT of the parotid gland is a rare neoplasm that occurs most often in middle-aged patients, without a gender predilection, who have symptoms of a mass lesion for a long duration. SFT is diagnosed based on classical histologic and positivity for CD34, Bcl-2, and STAT6 (nuclear pattern), which aid in its distinction from other tumors in the differential diagnosis. A short follow-up period suggests a good prognosis, whether the tumors are histologically benign or malignant when managed by complete surgical excision.

## Data Availability

The pictures used to support the findings of this study are included within the article and separated in figure files.
